# Pain Assessment in Patients Undergoing Maggot Debridement Therapy in the Process of Local Treatment of Chronic Wounds

**DOI:** 10.3390/jcm13030884

**Published:** 2024-02-02

**Authors:** Dariusz Bazaliński, Karol Sieńczak, Kamila Pytlak, Joanna Przybek-Mita, Klaudia Pelczar, Wojciech Leppert, Paweł Więch

**Affiliations:** 1 Institute of Health Sciences, College of Medical Sciences, University of Rzeszów, 35-959 Rzeszów, Poland; dbazalinski@ur.edu.pl (D.B.); kamila.pytlak@interia.pl (K.P.); joprzybek@ur.edu.pl (J.P.-M.); 2 Podkarpackie Specialist Oncology Centre, Father B. Markiewicz Specialist Hospital in Brzozów, 36-200 Brzozów, Poland; karolsienczak@up-sanok.edu.pl; 3 Institute of Medicine, Sanok State University, 38-500 Sanok, Poland; 4 Postgraduate Nursing and Midwifery Education Centre, 35-083 Rzeszów, Poland; 5 Independent Public Healthcare Institution, Ministry of Internal Affairs and Administration, 35-959 Rzeszów, Poland; pelczar.k@wp.pl; 6 Institute of Medical Sciences, Collegium Medicum, University of Zielona Góra, 65-046 Zielona Góra, Poland; w.leppert@inm.uz.zgora.pl; 7 University Clinical Hospital in Poznań, 60-245 Poznań, Poland

**Keywords:** pain intensity, maggot therapy debridement, nurse, wound

## Abstract

(1) Background: Developing and implementing strategies for local wound care focused on improving the quality of life related to health status and reducing treatment costs for this patient group poses a challenge to contemporary healthcare systems. The utilization of Maggot Debridement Therapy (MDT) is one potential form of local therapy for preparing wounds for the healing process. The debridement of the wound bed with medical maggots is highly precise, and the defensins produced by the larvae eliminate bacteria and stimulate tissue regeneration. However, the presence of larvae in the wound may lead to the occurrence of pain symptoms. The aim of the study was to assess the intensity of pain during larval therapy in patients with chronic wounds treated in outpatient settings. (2) Patients and Methods: The study employed a diagnostic survey and estimation; the tool consisted of a research protocol comprising three parts (questionnaires). Inclusion criteria for the study were voluntary consent to participate (completion of the MDT acceptance questionnaire), chronic wounds of vascular etiology or pressure injuries, full-thickness skin or deep tissue damage, and pain intensity not exceeding four on the NRS (Numerical Rating Scale: 0—no pain, 10—the most severe pain) at the time of the study. Patient observation during the 3-day treatment was conducted by a wound care clinic nurse, assessing pain intensity once every 24 h during the larval dressing changes. (3) Results: Out of 348 individuals who qualified for MDT during the study period, 215 individuals participated in the study: 94 women (43.7%) and 121 men (56.3%). The age of the participants ranged from 28 to 97 years (mean 69.87 ± 12.95). Each participant experienced mild pain (2.26 ± 1.60 on the NRS) on the day of qualification for the study. An increase in pain intensity, according to subjective assessments, was reported by 29.3% of participants (n = 63). On the third day of MDT therapy, an increase in pain intensity was observed, reaching a mean value of 4.79 ± 2.12 (*p* < 0.0001). Participants with pressure injuries showed the lowest pain intensity, which increased in consecutive days for all types of wounds. Additionally, the increase in pain intensity in patients with vascular etiology wounds was greater compared to patients with pressure injuries (*p* < 0.001). (4) Conclusions: Local wound therapy with *Lucilia sericata* larvae increases pain intensity in the consecutive days of treatment. The wound area and the time since its occurrence may determine pain symptoms.

## 1. Introduction

The rising incidence of chronic diseases, particularly diabetes and peripheral vascular diseases, is a primary cause of the prevalence of difficult-to-heal wounds in the population aged 60 and above [1,2], affecting 1–2% of the global population [3]. This poses a significant challenge to healthcare systems. Individuals with coexisting skin damage due to chronic vascular dysfunction exhibit a low quality of life related to health and a self-care deficit. The exacerbation of diseases, complications, self-care dysfunctions, often unprofessional care, and the COVID-19 pandemic have caused additional health problems. Prolonged treatment duration and frequent local complications (infections, pain, exudate, wound area enlargement) predispose patients to suffering, financial burdens, and challenges for patients, families, and the healthcare system. Developing and implementing wound care strategies aimed at improving health-related quality of life and reducing treatment costs are challenges for healthcare systems [4]. Self-care limitations, knowledge deficits, and implemented local therapies (compression therapy, negative pressure wound therapy—NPWT, and procedural therapeutic interventions within the wound) require professional care and specialized supervision, which can be provided by both physicians and nurses.

Local wound treatment is a crucial factor in ensuring professional and comprehensive care and treatment. Patient assessment, health diagnosis, and wound etiology recognition, as well as goal determination and directions for actions, provide the basis for implementing recommended therapeutic protocols. Wound bed preparation (WBP) in most cases is one of the first specialized activities undertaken as part of local therapy based on the TIME(RS) concept [5]. The selection of the optimal cleansing method depends on numerous factors, including the etiopathogenesis of the wound, coexisting diseases, the patient’s clinical condition, pain threshold, and the clinical experience of the healthcare provider, as well as the preferences and economic capabilities of the patient [6]. Wound bed preparation, involving the removal of necrotic tissue to enable the inspection of underlying tissues, includes the elimination of dead space, infection drainage, and optimization of wound edges and the wound bed to initiate tissue regeneration [7]. The optimal cleansing method should be based on the wound status, environmental factors, and the availability of medical staff. Surgical wound debridement (management) is the gold standard; however, not every wound qualifies for this method, which is reserved for physicians and often requires general anesthesia in an operating room setting. Wound debridement using small surgical instruments (scalpel, scissors, grasping tools) according to the TIME(RS) concept is termed sharp debridement and can be performed in a home, outpatient, or surgical setting, depending on the extent and progression of tissue destruction, by a qualified clinician (nurse or doctor) with specialized training and experience in wound prevention and treatment [8]. Mechanical debridement is the most basic form of wound decontamination using sponges or wound hygiene products. It is a crucial element that allows the modification of the non-physiological environment of a chronic wound to become that of an “acute wound”, stimulating natural repair processes connected with minimizing pro-inflammatory proteases and increasing keratinocyte and fibroblast migration. The sharp debridement of chronic wounds (removing necrotic slough) is possible and safest when tissue demarcation precedes wound debridement using maggot debridement therapy (MDT) [9,10].

*Lucilia sericata* larvae have been used in medicine since 2004, and are registered by the Food and Drug Administration (FDA) for therapeutic purposes. They liquefy and eliminate dead tissues, and stimulate repair processes by secreting waste and secretions with antibacterial, antibiofilm, and tissue regenerative properties into the wound. The concept of using MDT appears simple but requires engagement from caregivers, including adherence to the action protocol, pharmacological supply, and supervision of the therapy [10]. Quick and precise wound cleansing from devitalized tissue, minimizing bacteria in the wound, and stimulating repair processes related to MDT, compared to commonly used methods (active dressings and hydrogels), places this form of therapy in the area of interest for caregivers providing care and treatment for patients with chronic wounds [11,12]. MDT is associated with the risk of symptoms that can reduce the quality of life for patients. Patients often express reluctance due to the presence of larvae in the wound, associating them with poor hygiene, dirt, and spoiling food. The sight of moving maggots can negatively impact mental well-being and increase pain intensity [10]. Unpleasant somatic sensations, such as itching, foreign body sensation, and pain, are the main symptoms reported by patients during MDT therapy. Clarifying the mechanism of larvae action in the wound can help minimize patient discomfort [9,13]. The increase in pain intensity during MDT therapy should not be underestimated when striving for method standardization [9,10,11,14]. Pain intensity during MDT therapy should be minimized with simple first-line analgesics according to the World Health Organization’s analgesic ladder, considering that a wet dressing and the sight of larvae during dressing changes can be a cause of increased pain intensity [9,10,13,15]. Literature analysis and personal observations related to the intensity of perceived pain during MDT therapy prompted the conduct of a study aiming to assess pain intensity on different days of therapy. The study’s objective was to evaluate the intensity of pain during larval therapy among patients with chronic wounds treated in outpatient conditions.

## 2. Material and Methods

### 2.1. Ethical Considerations

The study protocol was approved by the Bioethics Commission at the University of Rzeszow (Resolution no. 30/06/2017, 30 June 2017). Moreover, the guidelines of the Helsinki Declaration were introduced in the course of the conducted study. Participants were informed about the purpose of the study, provided informed consent before starting the study, and could withdraw at any point without giving a reason. The conducted study did not have the characteristics of a medical experiment, and the authors do not report any conflicts of interest.

### 2.2. Subjects

**Figure 1:**
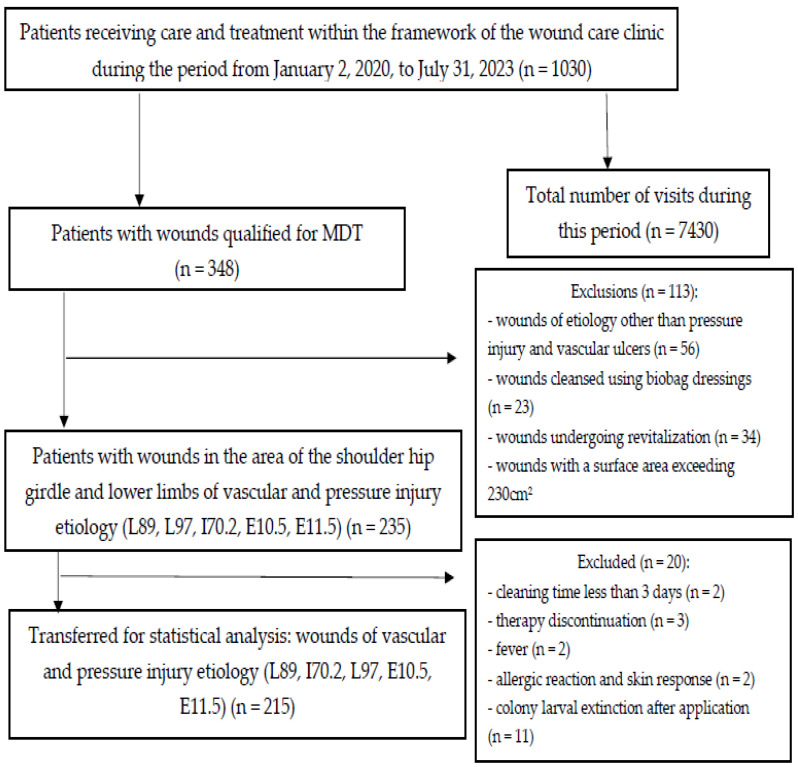
Study qualification protocol.

In a prospective observational study, patients undergoing treatment for chronic wounds located in the area of the pelvic girdle and lower limbs were included. The research was conducted at the Wound Treatment Clinic of the Podkarpackie Oncology Center in Brzozów, Poland, during the period from 2 January 2020, to 31 June 2023 (36 months). During this time, the clinic staff conducted over 7400 nurse-physician visits for 1030 individuals (Figure 1). Biological wound debridement was performed using 50 to 150 *Lucilia sericata* larvae (loose) from the Biolab^®^ breeding facility in Kędzierzyn Koźle, Poland.

### 2.3. Assessments

The research methodology involved a diagnostic survey coupled with estimation, and the research instrument comprised a protocol consisting of three parts (questionnaires). The first part pertained to the respondents’ sociodemographic data (age, gender, residence, marital status, self-care capacity according to Barthel). The second part comprised a wound assessment protocol conducted by the nurse (time of occurrence, etiology, appearance according to the RYB color classification (Red, Yellow, Blue), location, depth of damage to tissue structures (National Pressure Injury Advisory Panel, NPIAP) [8,16], and the classification of injuries within the foot (wound (W), ischemia (I), and foot infection (fI); WIFi) [17]. The third part included a protocol for assessing pain intensity (NRS, Numerical Rating Scale: 0—no pain, 10—the most severe pain), measured at the time of larval application and daily throughout the therapy (conducted by the nurse based on information obtained from the participant) [18].

The inclusion criteria for the study encompassed voluntary consent to participate in the study (completion of the MDT acceptance questionnaire). The tool comprises 10 questions, of which 2 are general inquiries related to coping with the challenges of living with a chronic wound. The remaining 8 questions specifically pertain to the patient’s feelings as a consequence of undergoing chronic wound therapy with *Lucilia sericata* larvae [13], chronic wounds of vascular etiology or pressure injuries, full-thickness skin damage (NPIAP 3°) or deep tissue damage (NPIAP 4°), wounds associated with diabetic foot disease (WIFi grade > 1°), and pain intensity not exceeding 4 on the NRS. The exclusion criteria encompassed patients who did not consent to participate in the research project and, based on the questionnaire assessment of MDT acceptance, scored less than 30 points. Patients with wounds of different etiology and location (post-traumatic, postoperative, atypical, tumor-related, located above the pelvic girdle and lower limbs), a larval therapy duration of less than 3 days, a lack of consent for this form of therapy, and pain intensity above 4 on the NRS were excluded from the study. In the case of multiple wounds, only one was assessed, which was qualified for MDT.

The differentiation of critical ischemia was performed using blind Doppler, and in the case of flow disturbances, a surgical duplex ultrasound scan of the lower limbs was conducted. Patients were also excluded from the study before revascularization if the wound showed no signs of infection and phlegmon (dry necrotic eschar).

### 2.4. Course of the Study

**Figure 2:**
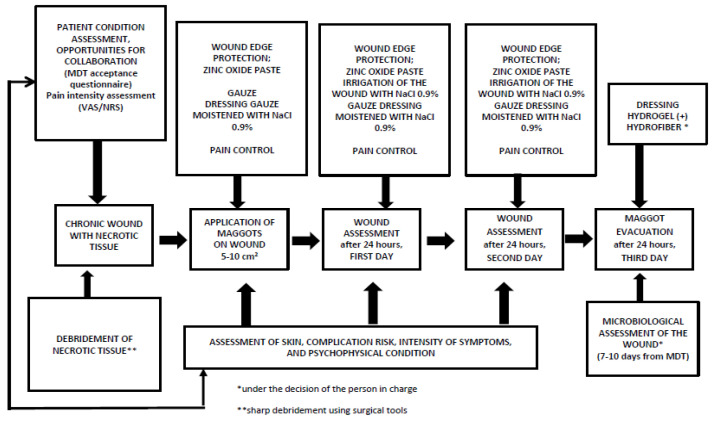
Larval application protocol and supervision of MDT therapy.

Patients who met the qualification criteria underwent a brief education session lasting several minutes regarding MDT, the principles of dressing observation, and communication with the person conducting the therapy. The procedural protocol was based on the guidelines of the Polish Wound Management Society (PTLR) [8,19] (Figure 2). The process of maintaining larvae in the wound lasted for 3 days (72 h). A designated individual with medical education and qualifications for wound treatment supervised the patient in person or using telecommunication systems (WhatsApp^®^, Messenger^®^). In the conducted study, loose larvae were applied at a preferred ratio of 5–10 larvae per cm^2^, with the surrounding skin typically protected using zinc paste. In some cases, apertures (enclosures) made of foam dressings and stoma paste, especially when compression therapy was necessary, were utilized. Compression therapy was not discontinued during MDT; instead, compression force was reduced to level 1 (15–25 mmHg). No adverse effects on the colonies were confirmed. Detailed actions and the therapy process are presented in Figure 2. Each patient was instructed regarding the possibility of using analgesic medications, mainly from the NSAIDs and non-opioid analgesic groups based on WHO guidelines. Individuals exhibiting signs of wound infection and those on continuous medication received instructions to increase the baseline medication dose by 25% in the first day of therapy, with the option of taking additional doses of non-opioid medications (paracetamol). Patients with neuropathic pain underwent individual preparation for MDT therapy.

### 2.5. Statistical Analysis

A total of 215 questionnaires were analyzed using the following methods: distribution analysis, arithmetic mean, median, standard deviation, Kolmogorov–Smirnov normality test, and Chi-square test. Differences in distributions were assessed using the Kruskal–Wallis test for more than two categories of the independent variable and the Mann–Whitney U test for comparing distributions in two categories of the variable. Friedman ANOVA was employed to evaluate differences in pain intensity measurements over four consecutive days. Post-hoc analysis to assess differences between specific measurements was conducted using the Wilcoxon signed-rank test. All calculations were performed using the IBM SPSS Statistics v. 21 (IBM Corp., Armonk, NY, USA), and a significance level of *p* < 0.05 was adopted. Abbreviations used in the tables: SD—standard deviation, df—degrees of freedom.

## 3. Results

### 3.1. Characteristics of the Respondents

A total of 215 individuals from the group of 348 eligible for MDT during the study period were included in the statistical analysis. The study involved 94 women (43.7%) and 121 men (56.3%). The participants’ ages ranged from 28 to 97, with a mean age of 69.87 ± 12.95 years. The largest age group consisted of individuals above 65 years (76.3%) (Table 1), with 153 (71.2%) residing in rural areas and 62 (28.8%) in urban areas. Self-care capabilities were limited in 66.5% of the studied group (n = 143); Barthel 21–85, with a total self-care deficit in 12.1% (n = 26); Barthel 0–20, and satisfactory in 21.4% (n = 42); Barthel 86–100. A low level of acceptance of MDT therapy (according to questionnaire assessment) was declared by 1.9% (n = 4) of individuals. The remaining participants showed average acceptance levels of 42.3% (n = 91) or high acceptance levels of 55.8% (n = 120).

### 3.2. Characteristics of Wounds Subjected to Maggot Debridement Therapy (MDT)

Based on the criteria for selecting patients for the study, it was confirmed that there were 52 pressure injuries (24.2%) and 162 wounds of vascular etiology (75.8%), including venous ulcers 30.7% (n = 66), mixed ulcers 25.6% (n = 55), arterial ulcers 10.7% (n = 23), and ulcers in the course of diabetic foot disease 8.8% (n = 19) (Table 1).

**Figure 3:**
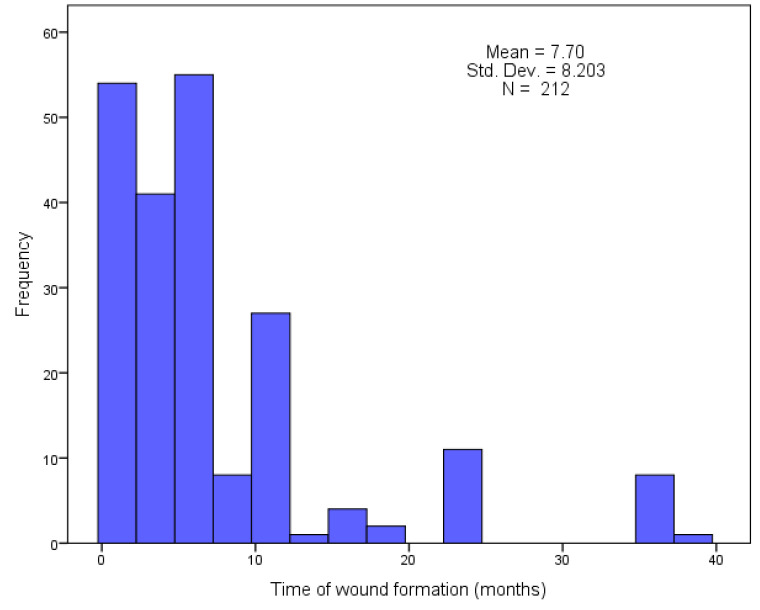
Histogram: Time from wound onset (n = 212).

The location of wounds varied, including the pelvic girdle, sacrum, trochanter, and ischial tuberosity 14.0% (n = 30), lower leg medial, anterior, posterior aspects 52.5% (n = 103), lower leg and foot area 3.7% (n = 51), and foot 8.8% (n = 19). Each wound met the criteria for a chronic wound (more than 6 weeks) with an average of 7.70 ± 8.203. The null hypothesis of the normal distribution of the time of wound occurrence was rejected, D (212) = 0.271, *p* < 0.001. Detailed data are presented in the histogram (Figure 3).

**Figure 4:**
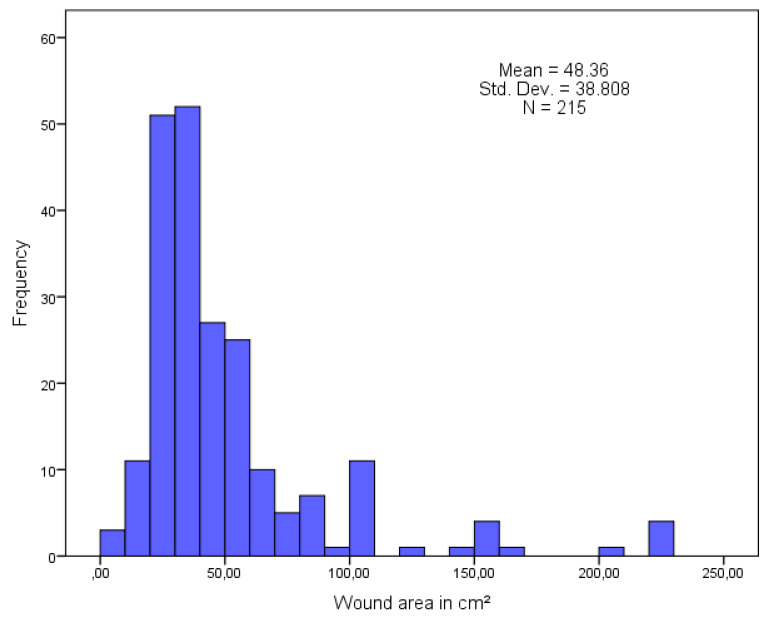
Histogram: Area of wounds undergoing MDT therapy (n = 215).

The area of tissue damage varied and ranged from 6 to 225 cm^2^, with a mean wound volume of 48.36 ± 38.808. The null hypothesis of a normal distribution of the wound area was rejected, D (215) = 0.251, *p* < 0.001. Detailed data are illustrated in the histogram (Figure 4).

Considering the evaluation according to the RYB color classification, wounds were predominantly yellow 58.1% (n = 125), red-yellow 32.1% (n = 69), and black 9.8% (n = 21). For the assessment of tissue destruction, several categorizations were applied according to the National Pressure Injury Advisory Panel (NPIAP): 3° (full skin thickness) 62.8% (n = 135), 4° (penetrating to the bone) 29.3% (n = 63). In the WIFi classification: 2° (in diabetic foot disease) 17.2% (n = 37), 3° 3.7% (n = 8).

### 3.3. Pain Intensity during the Three-Day MDT Therapy

Each of the study participants who qualified for the study reported minimal pain intensity. Out of 215 individuals, 28.4% (n = 61) were regularly taking analgesic medications (I or II on the WHO analgesic ladder). The number of individuals using analgesics (WHO I°) increased in the following days, reaching 56.3% (n = 121) on the third day. An increase in pain intensity was declared by 29.3% of patients (n = 63), and itching by 12.3% (n = 27) (*p* < 0.0001).

The pain intensity assessments were conducted four times: first at the larval application, followed by three measurements every 24 h. Each participant experienced mild pain intensity (2.26 ± 1.60 on the NRS) on the day of larval application. Throughout each day of therapy, an increase in pain intensity was observed, reaching a mean value of 4.79 ± 2.12 on the third day of MDT therapy (*p* < 0.0001) (Table 2 and Table 3).

### 3.4. Pain Intensity and Selected Variables

#### 3.4.1. Wound Type

The average pain intensity values were compared for the day of larval application and days 1, 2, and 3 for the examined wound types. For all types of wounds, there were statistically significant differences in pain intensity (*p* < 0.001) (Table 4).

**Figure 5:**
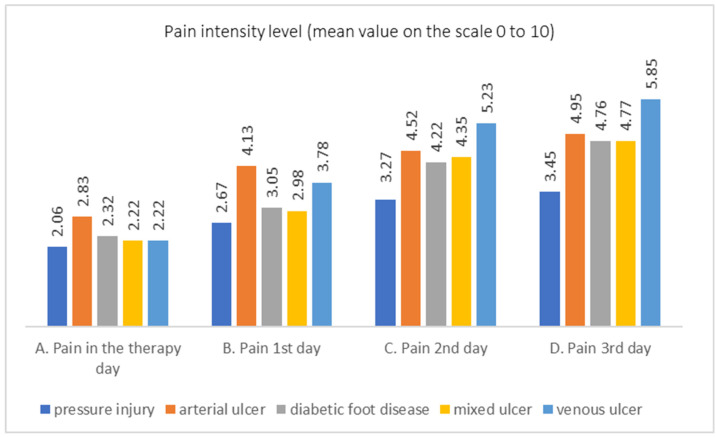
Pain intensity level on consecutive days for investigated wounds (pressure injuries and vascular etiology wounds).

For each type of wound, three comparisons of pain intensity measurements were conducted: between pain on the day of larval application and pain on day 1, between pain on day 1 and pain on day 2, and between pain on day 2 and pain on day 3. The results are presented in the chart (Figure 5). The order of wound types corresponds to the sum of differences in mean values between consecutive measurements. In the case of pressure injuries (the first wound type on the chart), the differences in mean pain intensity values were the smallest. Slightly larger differences in mean pain intensity values were observed between consecutive measurements in the case of arterial ulcers. Although, in the case of this wound type, the highest mean pain intensity values were observed on the day of larval application, the increase was smaller than in other types of wounds, such as diabetic foot ulcers, mixed ulcers, and venous ulcers. In the case of the last type of wound, the largest increases in pain intensity were observed on consecutive days, along with the highest overall pain intensity.

In the case of the therapy day, the null hypothesis should be accepted, stating that the distribution of pain intensity is the same for all types of wounds. However, for days 1, 2, and 3, these hypotheses should be rejected. Therefore, on the first, second, and third days, differences in pain intensity are observed depending on the type of wound (Table 5).

To check which distributions differ from each other, pairwise comparisons were made using the Mann–Whitney test. Nine comparisons were performed, so a significance level (of 0.05/9 = 0.006 for pairwise comparisons) was adopted. Since the result of the Kruskal–Wallis test indicates no dependency between pain intensity and the type of wound on the therapy day, it was examined how the level of pain intensity changes depending on the type of wound on the first, second, and third days. The results of the Mann–Whitney test (*p*-values) are presented in Table 6.

Significant differences in pain intensity were mainly observed on the third day, in the case of patients with pressure injuries compared to those with vascular-related wounds. Patients with pressure injuries experienced lower pain intensity. Considering that a conservative level of significance was adopted for the assessment of differences (*p* < 0.006, due to conducting nine pairwise comparisons), it can be inferred that there are differences in pain intensity among patients with different types of wounds. Pain intensity increased on consecutive days for all types of wounds, and the pain intensity was higher in patients with vascular-related wounds. The most significant differences occurred between the pain intensity in patients with pressure injuries and those with other types of wounds, especially venous and arterial ulcers (*p* < 0.001).

#### 3.4.2. Wound Area

**Figure 6:**
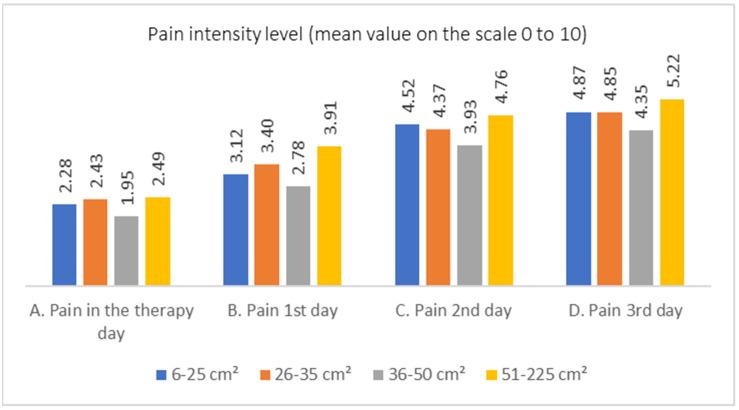
Pain intensity level over consecutive days for different wound sizes.

The hypothesis that a larger wound area corresponds to higher pain intensity during MDT was adopted. Regarding the assessment of pain intensity based on the wound area, the highest mean pain intensity values were observed in participants with a wound area exceeding 51 cm^2^ (Table 7). In Figure 6, pain intensity is graphically depicted based on the wound area subjected to MDT.

In the case of day 1, the hypothesis of equal distributions of mean pain intensity values across wound size categories should be rejected. On the first day, higher mean pain intensity values were observed among individuals with the largest wounds (51–235 cm^2^) compared to those with smaller wounds, with significant differences observed in the comparison between the largest wounds and moderately large wounds (36–50 cm^2^) (Table 8).

#### 3.4.3. Time since Onset of Wound

**Figure 7:**
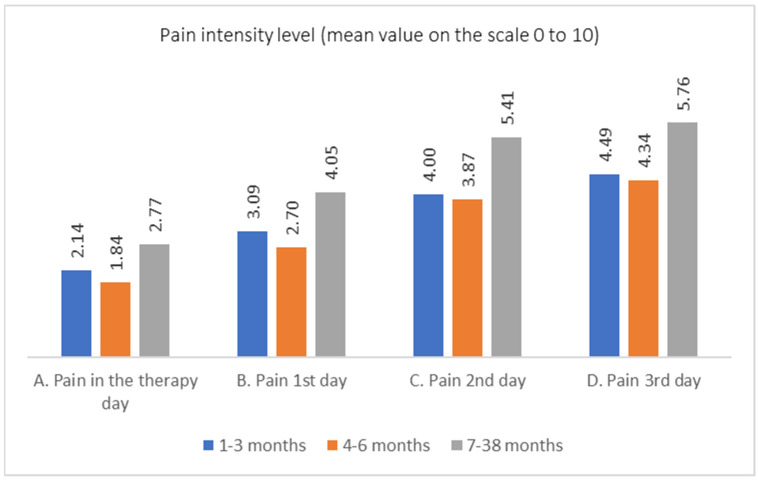
Pain intensity level on consecutive days for different wound sizes.

Each of the examined patients was undergoing treatment for a chronic wound that had occurred 4–8 weeks earlier. The majority of those undergoing MDT reported a time since the onset of the wound not exceeding 12 months. In assessing pain intensity within size-grouped categories of wounds, the highest average pain intensity values were observed in patients with wounds larger than 50 cm^2^ (Table 9, Figure 7).

For the therapy day as well as the first, second, and third days, the hypothesis of equal distributions of pain assessments in categories of wound creation time should be rejected. To examine which differences between pain distributions are significant, the Mann–Whitney test was conducted in three pairwise comparisons for each day of observation. No statistically significant differences were observed between pain assessments for wounds created 1–3 months before and those created 4–6 months before. For the therapy day, there was a significant difference between wounds created 1–3 months before and those created 7–38 months before. In all other cases, the differences were statistically significant. The data are presented in Table 10.

## 4. Discussion

In a prospective, observational study, 215 patients undergoing consultation and treatment at the Wound Treatment Clinic of the Podkarpackie Oncology Center in Brzozów were included. The clinic utilizes *Lucilia sericata* larvae in the local treatment process for debridement and tissue revitalization. The study focused on patients with chronic vascular etiology wounds and pressure injuries, aged 28 to 97 years (mean age 69.87 ± 12.95). Each patient experienced mild pain (2.26 ± 1.60 on the NRS) on the day of qualification for therapy. On the third day of maggot debridement therapy (MDT), moderate pain intensity was observed at 4.79 ± 2.12 (*p* < 0.0001). Patients with pressure injuries exhibited the lowest pain intensity, which increased in consecutive days for all wound types. Pain intensity increased each day of therapy. Patients with vascular etiology wounds reported higher pain intensity compared to those with pressure injuries (*p* < 0.001). Among all participants (n = 215), 61 individuals (28.4%) regularly took analgesics at the WHO’s I or II level. The number of individuals taking WHO I° analgesics increased in the following days, reaching 56.3% (n = 121) on the third day. Skin itching in the wound area occurred in 27 participants (12.3%), and pain intensity increased in 63 individuals (29.3%). Most participants undergoing MDT reported a time since wound occurrence of no more than 12 months. The hypothesis that a larger wound area corresponds to greater pain intensity during MDT was confirmed. In assessing pain intensity based on the wound area, the highest mean values were observed in individuals with a wound area above 51 cm^2^. No differences in pain intensity were observed on the first day for wounds occurring between 1–3 months and 4–6 months before. However, individuals with wounds of longer duration experienced higher pain intensity on the first day compared to those with shorter-duration wounds (*p* < 0.001).

Surgical debridement is considered the gold standard for the prompt management of wound beds, and it involves the direct removal of infected, necrotic, and devitalized tissues, coupled with a meticulous assessment of the depth of damaged structures. The objective of these interventions is to reduce the risk of local and systemic infections (SIRS) and to prepare the wound bed for the healing process. Surgical debridement, in clinically justified cases (traumatic wounds, burns, necrotizing fasciitis, abscess drainage, and hematomas) is conducted in operating rooms or surgical suites. This is known as “bloody debridement”, often necessitating sedation or local anesthesia [20]. It is crucial to differentiate between surgical procedures related to wound revision and the removal of infected and damaged tissue from acute management involving the use of instruments for debriding devitalized tissue (necrectomy). In the conducted study, skin destruction was assessed utilizing RYB, NPIAP, and WIfi classifications. As suggested by the authors, for the evaluation of tissue destruction in wounds of venous etiology, the Resvech classification should be considered. The utilization of systematized classifications will enable the standardization and potential harmonization of obtained results, facilitating comparisons with other reports [21,22]. The latter is a frequently performed specialized procedure in ambulatory care settings, designed to be a bloodless and safe intervention [23]. Unfortunately, access to medical personnel specializing in local wound therapy may be limited. Additionally, it should be noted that neither a surgeon nor a nurse can cleanse a wound with such precision as not to damage healthy tissue. Such meticulous debridement can only be achieved by the larvae of *Lucilia sericata*. Local therapy utilizing medical larvae is based on three mechanisms resulting from the larvae’s presence in the wound: mechanical and enzymatic cleansing of necrotic tissue, bactericidal and bacteriostatic actions, and support for the healing process, including physical contact with the wound area. Immature and non-reproductive larvae act as necrophages during their presence in the wound (3–5 days), undergoing three developmental stages. They actively and aggressively feed, assimilating approximately 25 mg of necrotic material from the wound within 24 h [10]. The larvae do not literally consume tissue, but they excrete and release digestive enzymes (digestive excretions and secretions, arginase). Digestion begins directly in the wound bed, outside the larva’s body. Necrotic tissue undergoes liquefaction and systemic absorption [23]. This phenomenon can be observed during therapy, as there is a significant increase in the amount of brownish exudate in the wound with a distinctive odor [13,14]. The analysis of wounds before and after the application of medical larvae demonstrates the promotion of wound healing on multiple fronts. Therefore, a significant aspect of MDT is its broad chemical action based on the secretion and excretion of specific enzymes, coupled with their correlation with antibacterial activity (Lucilin, Lucifensin, Lucifensin II), MAMP (Alpha–methoxyphenol), and seraticin [9,10,12,24].

Preparing the wound bed through the elimination of devitalized and necrotic tissues, along with reducing bacterial load, is a pivotal measure aimed at shortening the inflammatory phase and establishing conditions for optimal healing and tissue regeneration. The decision on the choice of wound cleansing method should be made considering the etiology, wound condition, available resources, and patient preferences [14]. MDT provides numerous benefits as a form of local therapy. Approximately 15,000 patients per year are qualified for local wound treatment using MDT [25,26]. In Poland, there is a lack of data in this area. In the center where the research was conducted, 7–10 individuals are qualified for MDT weekly. The most commonly observed adverse effects of therapy using medical larvae include itching, paresthesia (tingling), and pain [10,14,27,28]. Among the medical personnel, there is still a belief in the low efficacy and negative visual experiences, which cause some patients to not accept this method, fearing an increase in pain as well [9,14,15].

Most patients do not feel the presence of larvae (usually in diabetic neuropathy); some report a subjective sensation of movement in the wound, which does not cause discomfort. However, a portion of patients experience varying degrees of pain, for example, those with peripheral arterial disease. Our conducted studies represent the first scientific report on this matter in Poland. Understanding the issues related to pain intensity during local therapy will enable the preparation of guidelines for the optimal treatment of patients eligible for MDT. According to a retrospective analysis by Mumcuoglu et al., out of 435 patients treated for chronic wounds (totaling 723 wounds), 165 (38.0%) experienced either the occurrence or intensification of pain within the wound during the application of the bio-surgical dressing, which was maintained for a period of 3–5 days. In the majority of cases, pain was effectively alleviated with analgesics, and only in five patients was the therapy discontinued, and the dressings were removed [14]. In a randomized clinical trial conducted by Mudge et al., MDT significantly more rapidly cleansed ulcerative wounds than hydrogel. Individuals in the larval group experienced greater pain or discomfort associated with wound debridement compared to those with the traditional hydrogel dressing [11].

Pain, as an unpleasant subjective experience, is one of the distressing symptoms that often exhibits a complex pathophysiology (tissue damage, ischemia, neuralgia, infection, and psychological disorders) [29]. The increase in pain intensity may result from therapeutic procedures (including wound debridement, dressing changes, MDT). Pain associated with MDT is likely the result of larval movement on the wound surface, where they use two hooks to advance their bodies, and the outer layer of their bodies is covered with thorn-like bristles. Additionally, the excretions/secretions (ES) of the larvae, which include proteolytic enzymes, may affect exposed tissues and nerve endings within the wound area [30]. The assessment of pain intensity and perception should occur during health evaluation and physical examination. Following the physical examination, an assessment is conducted using an acceptance questionnaire (in Poland, the MDT acceptance questionnaire) [9,13]. Subsequently, education about MDT therapy and the principle of the three-day treatment is provided. Patients receive educational materials and provide written consent for larval application. Patients with diabetic foot disease may experience hypoalgesic-type sensory disturbances, and in this group, sensations during MDT are comparable to the pre-treatment period [31,32], whereas patients with ischemic ulcers may poorly tolerate MDT and experience hyperalgesia. These observations align with our own findings; however, our results were not statistically significant in the assessment of these variables. It is recommended to implement pharmacological interventions (pain relievers and co-analgesics) routinely before MDT treatment [9,10,14]. In our study, the least severe pain was observed in individuals with pressure injuries, while the highest pain intensity was observed in patients with lower leg ulcers of venous etiology. A limitation of our study is the lack of an assessment of infection characteristics in the wound, which could exacerbate pain experiences. In future studies, this variable should be considered as one of several factors related to pain intensity. It was noted that in the group of individuals with ischemic ulcers, pain was stronger than in other patient groups; however, in this group, patients adhered more closely to recommendations regarding the use of pain medication. Most participants in this group were disqualified from MDT if the pain intensity exceeded four on the NRS scale for subsequent preparation.

The use of medical maggots therapy is beneficial in the local treatment of wounds of various etiologies that meet the criteria for chronic non-healing wounds. The application of larvae can potentially reduce the overall cost of treatment, by either shortening or eliminating the need for hospitalization and decreasing antibiotic consumption. Complications associated with the use of biological debridement are rare and primarily involve psychosomatic experiences (pain, itching). Efforts should be made to develop algorithms that ensure optimal patient preparation for this form of therapy.

### Limitations

The study was conducted at a single center using a subjective pain intensity scale, despite the assessment of pain intensity being performed by the healthcare provider. Additionally, older individuals may have encountered difficulties in accurately assessing the intensity of pain. Despite recommendations to regularly take prescribed analgesic medications, only 121 participants adhered to the advice, with others considering analgesic use unnecessary. An assessment of wound infection was not conducted; participants exhibiting symptoms of infection may have presented with higher pain intensity. For wound assessment, the universal wound assessment system Resvech could have been used.

## 5. Conclusions

Approximately 30.0% of the participants reported an increase in pain intensity in subjective assessments. Local wound therapy with *Lucilia sericata* larvae results in an elevation of pain intensity in the consecutive days following larval application. Individuals with vascular etiology wounds experienced greater pain intensity compared to those with pressure injuries and diabetic foot disease. Both the wound area and the time since its occurrence may influence the intensity of pain.

## Figures and Tables

**Table 1:** Type of injury and level of tissue destruction (n = 215).

		n	%
Venous ulcers		66	30.7%
Mixed ulcers		55	25.6%
Pressure injuries		52	24.2%
Arterial ulcers		23	10.7%
Ulcers in the course of diabetic foot disease		19	8.8%
Total		215	100.0%
Wound assessment (RYB)	Yellow	125	58.1%
	Red/yellow	66	30.7%
	Black	24	10.2%
Wound assessment (NPIAP)	3° (full skin thickness)	152	70.7%
	4° (penetrating to the bone)	63	29.3%
	Total	215	100.0%
Wound assessment (Wifi)	II	17	89.5%
	III	2	10.5%
	Total	19	100.0%

**Table 2:** Descriptive statistics of pain intensity according to NRS (n = 215).

	Mean	SD	Median	Min	Max	25th Perc.	75th Perc.	n
A. Pain intensity on the day of larval application	2.26	1.60	2	1	4	1	3	204
B. Pain intensity 1st day	3.26	1.96	3	1	6	2	4	206
C. Pain intensity 2nd day	4.37	2.16	4	1	10	3	6	204
D. Pain intensity 3rd day	4.79	2.12	4	1	10	3	6	191

**Table 3:** Normality tests (n = 215).

	Kolmogorov-Smirnov		
	Statistics	df	Significance
A. Pain intensity on the day of larval application	0.275	204	0.0001
B. Pain intensity 1st day	0.213	206	0.0001
C. Pain intensity 2nd day	0.200	204	0.0001
D. Pain intensity 3rd day	0.147	191	0.0001

The variables with pain ratings on a scale from 0 to 10 do not have a normal distribution.

**Table 4:** Pain intensity ratings on consecutive days depending on wound type (n = 215) (Friedman ANOVA test).

Statistics	Pressure Injury	Mixed Ulcer	Arterial Ulcer	Venous Ulcer	Diabetic Foot Disease
n	52	47	21	53	17
F	83.93	97.10	38.76	128.36	35.70
df	3	3	3	3	3
*p*	0.0001	0.0001	0.0001	0.0001	0.0001

**Table 5:** Distribution of pain intensity ratings by wound type categories (n = 215) (Kruskal–Wallis test).

	A. Pain on the Therapy Day	B. Pain 1st Day	C. Pain 2nd Day	D. Pain 3rd Day
Chi-square	6.141	13.495	25.725	36.263
df	4	4	4	4
Asymptotic significance	0.189	0.009	0.0001	0.000

**Table 6:** Assessment of pain intensity on individual days depending on the type of wound (n = 215) (Mann–Whitney test).

1st day	Mixed Ulcer	Arterial Ulcer	Venous Ulcer	Diabetic Foot Disease
Pressure injury	0.375	0.001	0.008	0.392
Mixed ulcer		0.015	0.122	0.893
Arterial ulcer			0.204	0.050
Venous ulcer				0.278
2nd day	Mixed ulcer	Arterial ulcer	Venous ulcer	Diabetic foot disease
Pressure injury	0.009	0.002	0.000	0.134
Mixed ulcer		0.486	0.025	0.752
Arterial ulcer			0.233	0.392
Venous ulcer				0.064
3rd day	Mixed ulcer	Arterial ulcer	Venous ulcer	Diabetic foot disease
Pressure injury	0.001	0.001	0.000	0.020
Mixed ulcer		0.711	0.010	0.899
Arterial ulcer			0.074	0.520
Venous ulcer				0.028

**Table 7:** Pain intensity assessment in relation to wound area (n = 215).

		Wound Area			
		6–25 cm^2^	26–35 cm^2^	36–50 cm^2^	51–225 cm^2^
A. Pain intensity on the day of therapy	Mean	2.28	2.43	1.95	2.49
	SD	1.87	2.09	1.11	1.21
	Median	2	2	2	2
	Percentile 25	1	1	1	2
	Percentile 75	3	3	2	3
	n	58	40	59	47
B. Pain intensity 1st day	Mean	3.12	3.4	2.78	3.91
	SD	1.97	2.27	1.46	2.04
	Median	3	3	3	3
	Percentile 25	2	2	2	3
	Percentile 75	4	5	4	4
	n	58	42	59	47
C. Pain intensity 2nd day	Mean	4.52	4.37	3,93	4.76
	SD	2.36	2.26	1.79	2.21
	Median	4	4	4	4
	Percentile 25	3	3	3	3
	Percentile 75	6	6	5	6
	n	58	41	60	45
D. Pain intensity 3rd day	Mean	4.87	4.85	4.35	5.22
	SD	2.26	2.16	1.79	22.26
	Median	5	4	4	5
	Percentile 25	4	3	3	4
	Percentile 75	6	7	5	6
	n	54	39	57	41

**Table 8:** Assessment of pain intensity in relation to wound area (n = 215) (Kruskal–Wallis test).

	A. Pain Intensity on the Day of Larval Application	B. Pain Intensity 1st Day	C. Pain Intensity 2nd Day	D. Pain Intensity 3rd Day
Chi-square	7.076	9.089	4.203	3.448
df	3	3	3	3
Asymptotic significance	0.070	0.028	0.240	0.328

**Table 9:** Pain intensity assessment based on the time of wound occurrence (n = 215).

		Time of Wound Occurrence		
		1–3 Months	4–6 Months	7–38 Months
A. Pain intensity on the day of therapy	Mean	2.14	1.84	2.77
	SD	1.38	1.13	2.05
	Median	2	2	2
	Percentile 25	1	1	1
	Percentile 75	3	2	4
	n	76	62	64
B. Pain intensity 1st day	Mean	3.09	2.7	4.05
	SD	1.64	1.62	2.39
	Median	3	3	4
	Percentile 25	2	1	3
	Percentile 75	4	3	5
	n	77	63	63
C. Pain intensity 2nd day	Mean	4	3.87	5.41
	SD	1.81	1.96	2.44
	Median	4	3	5
	Percentile 25	3	3	4
	Percentile 75	5	5	7
	n	78	63	61
D. Pain intensity 3rd day	Mean	4.49	4.34	5.76
	SD	1.78	2.17	2.21
	Median	4	4	6
	Percentile 25	3	3	4
	Percentile 75	5	5	7
	n	74	61	54

**Table 10:** Pain intensity assessment on specific days based on the time of wound occurrence (n = 212) (Mann–Whitney test).

The Day of Therapy	4–6 Months	7–38 Months
1–3 months	0.146	0.080
4–6 months		0.004
1st day	4–6 months	7–38 months
1–3 months	0.113	0.014
4–6 months		0.000
2nd day	4–6 months	7–38 months
1–3 months	0.542	0.000
4–6 months		0.000
3rd day	4–6 months	7–38 months
1–3 months	0.463	0.001
4–6 months		0.000

## Data Availability

The mentioned authors consented to the publication of the research material.

## References

[1] Martinengo L., Olsson M., Bajpai R., Soljak M., Upton Z., Schmidtchen A., Car J., Järbrink K. (2019). Prevalence of chronic wounds in the general population: Systematic review and meta-analysis of observational studies. Ann. Epidemiol..

[2] Olsson M., Järbrink K., Divakar U., Bajpai R., Upton Z., Schmidtchen A., Car J. (2019). The humanistic and economic burden of chronic wounds: A systematic review. Wound Repair Regen..

[3] Järbrink K., Ni G., Sönnergren H., Schmidtchen A., Pang C., Bajpai R., Car J. (2016). Prevalence and incidence of chronic wounds and related complications: A protocol for a systematic review. Syst. Rev..

[4] Pytlak K., Szymańska P., Skórka M., Bazaliński D. (2023). Quality of life of patients covered by the Complex Treatment of Chronic Wounds. Surg. Vasc. Nurs..

[5] Atkin L., Bućko Z., Montero E.C., Cutting K., Moffatt C., Probst A., Romanelli M., Schultz G.S., Tettelbach W. (2019). Implementing TIMERS: The race against hard-to-heal wounds. J. Wound Care.

[6] Cwajda-Białasik J., Mościcka P., Szewczyk M. (2019). Selected methods of treatment of chronic wounds. Surg. Vasc. Nurs..

[7] Harries R.L., Bosanquet D.C., Harding K.G. (2016). Wound bed preparation: TIME for an update. Int. Wound J..

[8] Szewczyk M., Cwajda-Białasik J., Mościcka P., Cierzniakowska K., Bazaliński D., Jawień A., Spannbauer A., Polak A., Sopata M., Kozłowska E. (2020). Treatment of pressure ulcers—Recommendations of the Polish Wound Management Association. Part II. Leczenie Ran.

[9] Bazaliński D., Przybek-Mita J., Pytlak K., Kardyś D., Bazaliński A., Kucharzewski M., Więch P. (2023). Larval Wound Therapy: Possibilities and Potential Limitations—A Literature Review. J. Clin. Med..

[10] Sherman R.A. (2014). Mechanisms of Maggot-Induced Wound Healing: What Do We Know, and Where Do We Go from Here?. Evid. Based Complement. Altern. Med..

[11] Mudge E., Price P., Neal W., Harding K.G. (2014). A randomized controlled trial of larval therapy for the debridement of leg ulcers: Results of a multicenter, randomized, controlled, open, observer blind, parallel group study. Wound Repair Regen..

[12] Sun X., Chen J., Zhang J., Wang W., Sun J., Wang A. (2016). Maggot debridement therapy promotes diabetic foot wound healing by up-regulating endothelial cell activity. J. Diabetes Its Complicat..

[13] Bazaliński D. (2019). Efficacy of Biological Therapy using Lucilia sericata Larvae in the Treatment of Chronic Wounds in Patients in Long-Term and Palliative Care.

[14] Mumcuoglu K., Davidson E., Avidan A., Gilead L. (2012). Pain related to maggot debridement therapy. J. Wound Care.

[15] Moya-López J.M., Costela-Ruiz V., García-Recio E.M., Sherman R.A.M., De Luna-Bertos E. (2020). Advantages of Maggot Debridement Therapy for Chronic Wounds: A Bibliographic Review. Adv. Ski. Wound Care.

[16] Kottner J., Cuddigan J., Carville K., Balzer K., Berlowitz D., Law S., Litchford M., Mitchell P., Moore Z., Pittman J. (2019). Prevention and treatment of pressure ulcers/injuries: The protocol for the second update of the international Clinical Practice Guideline 2019. J. Tissue Viability.

[17] Monteiro-Soares M., Russell D., Boyko E.J., Jeffcoate W., Mills J.L., Morbach S., Game F. (2020). International Working Group on the Diabetic Foot (IWGDF) Guidelines on the classification of diabetic foot ulcers (IWGDF 2019). Diabetes/Metab. Res. Rev..

[18] Wikström L., Nilsson M., Broström A., Eriksson K. (2019). Patients’ self-reported nausea: Validation of the Numerical Rating Scale and of a daily summary of repeated Numerical Rating Scale scores. J. Clin. Nurs..

[19] Mrozikiewicz-Rakowska B., Tusiński M., Lipiński P., Bazaliński D., Dynarska J., Czwakiel L., Zymon A., Mospan B., Malinowska K., Sopata M. (2023). Statement of the Polish Wound Management Association on larval therapy in wound management. Leczenie Ran.

[20] Sibbald R.G.M., Elliott J.A.M., Persaud-Jaimangal R.M., Goodman L.M., Armstrong D.G.D., Harley C.R., Coelho S.B., Xi N.M., Evans R.M., Mayer D.O.M. (2021). Wound Bed Preparation 2021. Adv. Ski. Wound Care.

[21] Restrepo-Medrano J.C., Soriano J.V. (2011). Desarrollo de un índice de medida de la evolución hacia la cicatrización de las heridas crónicas. Gerokomos.

[22] Menegon M.R., Malaquias S.G., da Silva J.A., de Oliveira B.G.R.B., Medrano J.C.R., Verdú-Soriano J., Bachion M.M. (2023). RESVECH 2.0: Cross-cultural adaptation for Brazil, reliability and validity for the evaluation of venous ulcers. Rev. Bras. Enferm..

[23] King C. (2020). Changing attitudes toward maggot debridement therapy in wound treatment: A review and discussion. J. Wound Care.

[24] Gazi U., Taylan-Ozkan A., Mumcuoglu K.Y. (2021). The effect of *Lucilia sericata* larval excretion/secretion (ES) products on cellular responses in wound healing. Med. Veter.-Entomol..

[25] Nigam Y. (2021). The principles of maggot therapy and its role in contemporary wound care. Nurs. Times.

[26] Cazander G., Pritchard D.I., Nigam Y., Jung W., Nibbering P.H. (2013). Multiple actions of *Lucilia sericata* larvae in hard-to-heal wounds: Larval secretions contain molecules that accelerate wound healing, reduce chronic inflammation and inhibit bacterial infection. BioEssays.

[27] Morozov A.M., Sherman R.A. (2019). Survey of patients of the Tver region of Russia regarding maggots and maggot therapy. Int. Wound J..

[28] Hopkins R.C., Williams S., Brown A., Humphreys I., Clifford R., Nigam Y. (2022). Evaluating nursing opinion and perception of maggot therapy for hard-to-heal wound management. J. Wound Care.

[29] Eriksson E., Liu P.Y., Schultz G.S., Martins-Green M.M., Tanaka R., Weir D., Gould L.J., Armstrong D.G., Gibbons G.W., Wolcott R. (2022). Chronic wounds: Treatment consensus. Wound Repair Regen..

[30] Chambers L., Woodrow S., Brown A., Harris P., Phillips D., Hall M., Church J., Pritchard D. (2003). Degradation of extracellular matrix components by defined proteinases from the greenbottle larva *Lucilia sericata* used for the clinical debridement of non-healing wounds. Br. J. Dermatol..

[31] Zarchi K., Jemec G.B. (2012). The efficacy of maggot debridement therapy—A review of comparative clinical trials. Int. Wound J..

[32] Chadwick P., McCardle J., Ricci E., Stang D., Vig S. (2015). Appropriate use of larval debridement therapy in diabetic foot management: Consensus recommendations. Diabet. Foot J..

